# Evaluation of HER2 Positivity Based on Clinicopathological Findings in HER2 Borderline Tumors in Iranian Patients with Breast Cancer 

**DOI:** 10.30699/IJP.2023.561915.2970

**Published:** 2023-10-15

**Authors:** Ramesh Omranipour, Newsha Nazarian, Sadaf Alipour, Alireza Abdollahi, Bita Eslami

**Affiliations:** 1 *Breast Diseases Research Center, Cancer Institute, Tehran University of Medical Sciences, Tehran, Iran*; 2 *Department of Surgical Oncology, Cancer Institute, Tehran University of Medical Sciences, Tehran, Iran*; 3 *Islamic Azad University, Tehran Medical Sciences branch, Tehran, Iran*; 4 *Department of Surgery, Arash Women’s Hospital, Tehran University of Medical Sciences, Tehran, Iran*; 5 *Department of Pathology, School of Medicine, Imam Khomeini Hospital, Tehran University of Medical Sciences Tehran, Iran*

**Keywords:** Breast Cancer; HER2, Hybridization in Situ

## Abstract

**Background & Objective:**

Human epidermal growth receptor-2 (HER2) gene amplification is an important predictive and prognostic factor in breast cancer treatment. However, the expression of HER2 determined by immunohistochemistry (IHC) is considered as borderline in some cases, and confirmation of the HER2 status by either fluorescent in situ hybridization (FISH) or chromogenic in situ hybridization (CISH) is necessary for correct treatment decision-making. Considering the high cost of FISH and CISH, we aimed to investigate whether clinicopathological findings of the tumor could predict the HER2 status.

**Methods:**

A retrospective study was performed using the data from 584 patients with breast cancer with HER2-borderline disease, confirmed by IHC. Final HER2 status, pathologic tumor size and type, nodal involvement, Ki67 index, presence of estrogen and progesterone receptors (ER, PR), lymphovascular invasion (LVI), and stage were retrieved from the clinical records.

**Results:**

One hundred twenty-one (20.7%) patients were HER2-positive according to the FISH or CISH results. Logistic regression analysis showed that the pathologic size was positively associated with HER2 positivity with an odds ratio (OR) of 1.02 (95% CI: 1.01-1.04). In addition, the adjusted OR illustrated a statistically significant association between HER2 positivity and PR negativity (OR= 2.22, 95% CI: 1.29-3.83).

**Conclusion:**

In HER2 borderline breast cancer, HER2 positivity significantly increases with tumor size and PR negativity. Further studies are recommended that may find an applicable model to predict the actual status of HER2 in borderline cases.

## Introduction

In usual circumstances, human epidermal growth receptor-2 (HER2) plays an important role in normal cell growth and differentiation. However, amplification of the *HER2* gene leads to the overexpression of the receptor, thereby resulting in the development of many types of cancers including breast cancer (1). HER2 amplification was reported in 15% to 20% of breast cancers (2). A systematic review from Iran with significant heterogeneity among the included articles showed that the rate of HER2-positive breast cancers varied from 23.3% to 81% (3). Several studies have suggested that the HER2 subtype of breast cancer is associated with an aggressive course, higher relapse and mortality rate, and reduced levels of estrogen and progesterone receptors (4, 5). A separate study by Kadivar *et al.* reported 11.9% as the prevalence of HER2 subtype in Iranian women with breast cancer. They also showed vascular invasion and higher-grade tumors to be more prevalent in this subtype of cancer (6). Moreover, regardless lymph node involvement, survival analysis has shown that HER2 amplification would be the best predictive factor for the clinical outcome (7). 

Roses and co-workers showed that although high nuclear grade, large lesion size, and HER2 overexpression in ductal carcinoma *in situ* (DCIS) were associated with invasive disease on univariate analysis, HER2 is the only significant predictor for the presence of invasive breast cancer. Therefore, targeting HER2 in an early stage of the disease might prevent disease progression (8). Thus, a precise HER2 test result is necessary for accurate prediction of the disease progression before any anti-HER2 therapy. 

In routine practice, expression of HER2 is determined by immunohistochemistry (IHC) as +1 (negative) and +3 (positive). Complete intense membranous staining (more than 10%) is defined as a positive result. However, some cases (scored +2 for HER2) are considered as borderline tumors that cannot be classified as HER2 positive or negative. Either fluorescent in situ hybridization (FISH) or chromogenic in situ hybridization (CISH) is recommended in borderline cases to confirm the presence of *HER*2 gene amplification (7). Dual probe FISH analysis remains the most useful test which should be applied in all cases when immunostaining is doubtful or has a technical artifact (9). 

In our country, IHC is the initial step for HER2 detection. HER2 borderline tumors are further assayed via either FISH or CISH tests, which are expensive and time-consuming, and also unavailable in many centers. In some cases, due to the economic issues, further evaluation with FISH is not performed or may be postponed. For these patients, developing a predictive model that could properly estimate the result of FISH in HER2-borderline breast cancer would be very useful and prevent treatment delays. Therefore, this study was designed to determine the prevalence of HER2 positivity in HER2 borderline tumors in Iranian patients with breast cancer and to study correlation of the histopathologic characteristics of the tumor with the HER2 positivity. 

## Material and Methods

This retrospective cross-sectional study was conducted in accordance with the Declaration of Helsinki and the Institutional Research Board (No# 97-03-218-40456), and the Ethics Committee of Tehran University of Medical Sciences approved the study (IR.TUMS.VCR.REC.1397.890). The selected records belonged to all breast cancer patients who had been attending one private clinic between 2010 and 2020 (for 10 years) and had a borderline HER2 status. All the patients who had the results of FISH or CISH in their records were included in the study for final analysis. The clinicopathological characteristics including patient age, family history, laterality of the tumor, tumor grade according to the modified Bloom-Richardson classification, presence of lymphovascular invasion (LVI), pathologic tumor size and type, presence of distant metastases and disease stage, nodal involvement, Ki67 index, estrogen receptor (ER), progesterone receptor (PR), and final HER2 status were retrieved from the clinical records. 

The SPSS software (SPSS, Version 20, SPSS, Inc., IL., USA) was used for statistical analyses. The differences in means was tested using Student’s t-test in HER2 positive and negative cases. Categorical variables were compared by the Chi-square (χ^2^) test. A two-sided P-value of less than 0.05 was considered as statistically significant. Multivariable binary logistic regression was performed to estimate the odds ratio (OR) and confidence interval (CI) for the association between histopathologic variables and the HER2 status. Variables were selected a priori for inclusion in the multivariable model on the basis of the association with HER2 status in univariable analyses (*P*<0.1) and possible association in the literature.

## Results

Among the patients whose IHC had revealed HER2 borderline disease, 584 had FISH or CISH results in their records and were included in the final analysis. The mean age of the patients was 50.16±12.10, ranging from 23 to 83 years. Table 1 represents the tumor characteristics of all the breast cancers in the study population. Family history was positive in 186 (31.8%) cases.

In the total population, 27 cases showed metastasis and the most common sites of metastases were lung, bone, brain, and liver. One hundred twenty-one (20.7%) patients had final positive results for HER2 and the remaining (n=463, 79.3%) had negative results. Table 2 compares the tumor and patient characteristics of the cases with positive and negative HER2 status. Our results showed that larger tumor size and PR negativity were more prevalent in HER2-positive cases. The results of the logistic regression analysis considering pathologic tumor size and type, Ki67 index (<15% and ≥ 15%), metastasis, and PR as independent variables are shown in Table 3. HER2 positivity had a significant association with pathologic tumor size, having an odds ratio (OR) of 1.01 (95% CI: 1.01-1.04, *P*=0.02). In addition, the adjusted OR illustrated a statistically significant association between HER2 positive and PR negative (OR=2.22, 95% CI: 1.29-3.83, *P*=0.004) features. Contrarily, the invasive lobular carcinoma (ILC) showed a reverse association of borderline significance level (OR=0.25, 95% CI: 0.06-1.08, *P*=0.06) with HER2 positive status, only in univariate analysis. None of the other variables showed any association with HER2 status.

**Table 1:** Total characteristics of the study population (n = 584)

Age (yrs)	50.16 ± 12.10 (range: 23-83)
Pathologic Tumor size (mm)	26.64 ± 14.76 (1-130)
Ki67%	27.15 ± 19.36 (1-90)
Laterality Right Left	311 (53.3) 273 (46.7)
Node Involvement Positive Negative Unknown	250 (42.8) 316 (54.1) 18 (3.1)
Metastasis Yes No	27 (4.6) 557 (95.4)
Tumor Grade 1 2 3 Missing	41 (7) 350 (59.9) 142 (24.3) 51 (8.7)
Lymphovascular Invasion (LVI) No Yes Unknown	240 (41.1) 261 (44.7) 83 (14.2)
Breast Cancer Type IDC ILC Missing	497 (85.1) 31 (5.3) 56 (9.6)
Hormone Receptor ER + PR +	488 (83.6) 437 (74.8)
Method of determination HER2 status FISH CISH	421 (72.1) 163 (27.9)

Data are presented as mean ± Standard deviation and number (percentage), when appropriate. IDC: invasive ductal carcinoma; ILC: invasive lobular carcinoma; ER: estrogen receptor, PR: progesterone receptor.

**Table 2:** Comparison of variables between HER2 positive and negative patients

Variable	HER2 Positive (n=121)	HER2 Negative (n=463)	P-value
Age	50.53± 12.28	48.71 ± 11.31	0.14
Pathologic Tumor Size < 10mm ≥ 10mm	23.84 ± 13.24 2 (2) 98 (98)	28.06 ± 19.73 34 (7.9) 396 (92.1)	0.01 0.04
Ki67% < 15 ≥ 15	26.13 ± 19.28 24 (23.1) 80 (76.9)	31.43 ± 19.19 119 (27.5) 314 (72.5)	0.01 0.39
Breast Cancer Type IDC ILC	106 (98.1) 2 (1.9)	391 (93.1) 29 (6.9)	0.05
Positive family history of breast/ovarian cancer Yes No	36 (29.8) 85 (70.2)	150 (32.4) 313 (67.6)	0.58
Node Involvement Yes No	54 (45.8) 64 (54.2)	196 (43.8) 252 (56.3)	0.70
Metastasis Yes No	8 (6.6) 113 (93.4)	19 (4.1) 444 (95.9)	0.24
Tumor Grade 1 2 3	8 (7.7) 61 (58.7) 35 (33.7)	33 (7.7) 289 (67.4) 107 (24.9)	0.19
Lymphovascular invasion (LVI) Positive Negative	52 (51) 50 (49)	209 (52.4) 190 (47.6)	0.80
ER Positive Negative	95 (80.5) 23 (19.5)	393 (85.4) 67 (14.6)	0.19
PR Positive Negative	80 (68.4) 37 (31.6)	357 (78.5) 98 (21.5)	0.02

IDC = invasive ductal carcinoma; ILC = invasive lobular carcinoma; ER = estrogen receptor; PR = progesterone receptor.

**Table 3:** Results of the univariable and multivariable analysis considering HER2 status as a dependent variable

	Univariable Analysis		Multivariable Analysis	
	**Crude OR (95%CI)**	**P-value**	**Adjusted OR (95%CI)**	**P-value**
Pathologic size	1.02 (1-1.03)	0.01	1.02 (1.01-1.04)	0.02
Ki67% (≥15/ <15)	1.26 (0.76-2.09)	0.36	1.11(0.62-1.98)	0.74
Metastasis (Yes/No)	1.65 (0.71-3.88)	0.25	0.63 (0.17-2.34)	0.49
PR (Negative/Positive)	1.69 (1.08-2.64)	0.02	2.22 (1.29-3.83)	0.004
Breast Cancer Type (ILC/IDC)	0.25 (0.06-1.08)	0.06	0.001 (-)*	0.99

Variables were entered into the multivariable models based on p-value in univariate analysis (p-value <0.15). Therefore, pathologic size (mm), Ki67 category (≥ 15/ <15,), surgical pathology (IDC and ILC), metastasis, and PR (Negative/Positive) were entered in the model.

*Due to low number of positive ILC (n=2) the result of multivariable analysis was not significant.

## Discussion

In this study, breast cancers with borderline HER2 status were evaluated in Iranian patients, and we sought to develop a predictive model for the estimation of the actual HER2 status in these cases. In the present study, the prevalence of HER2 amplification was 20.7% (121 out of 584) in patients with IHC-based borderline HER2 results. Our results revealed a positive association between a final HER2-positive status and tumor size as well as with PR negativity. In ILC patients, two of them (6.5%) who had pleomorphic ILC had the positive HER2 result detected by FISH.

Although the prevalence of HER2 positivity in borderline HER2 patients in our study was higher than that of a large cohort of patients in India, which was estimated as 14.6% (5), in numerous studies, the rate of HER2 amplification confirmed by FISH in IHC borderline tumors was higher than that in the present study, from 27.5% to 70 % (10-14). The reason for such a high reported rate has been explained in some of the publications. One study by Okaly *et al.* in 2019 reported that more than half of the patients (54%: 72 out of 134) with borderline HER on IHC had HER2 amplification on FISH due to a possible referral bias (15). Similarly, Panjwani *et al. *explained the high rate of HER2 IHC in borderline cases which was 66.6% (24/36) with a high load of referral cases, quality of tissue fixation, method of processing, and duration of storage (16). In fact, some of these variabilities could be justified by inter-observer and intra-observer variations in IHC interpretation and the evolution of HER2 practice guidelines. 

Of note, all of these studies were conducted before the update of the American Society of Clinical Oncology (ASCO)/ College of American Pathologists (CAP) practice guideline in 2018, and application of this guideline may decrease the rate of HER2 positivity (17). Wei *et al.* studied the quantitative impact of 2018 ASCO/ CAP guidelines on HER2 status and showed an average of 9% reclassification in overall HER2 status with a net increase in HER2- negative designation (18).

The present study found an association between HER2 amplification and tumor size. Limited studies have evaluated the association between tumor size and HER2 amplification by FISH/CISH in cases with borderline IHC. Taucher *et al.* found that tumor size and HER2 status were inversely associated. In their study, 32.8% (n=22) of 67 patients with tumors larger than 5 cm were HER2 positive (19). In contrast, another investigation by Prati *et al.* did not find any association between HER2 status determined by FISH and tumor size as well as node status, presence of LVI, and patient age (10). Only tumor grade, P53 positivity, and negative hormone receptors had an association with HER2 positivity in the study of Prati *et al.* The difference between these and our results may be explained by the larger size of tumors in our patients which is due to the absence of a breast cancer screening program in our country. The average tumor size in cases of our study was 26.64 ± 14.76 mm which is certainly higher than that of other studies.

Our study showed an association between PR-negative and HER2-positive characteristics in HER2 patients with borderline results on IHC. Several studies have been conducted about the association of HER2 amplification by FISH and hormone receptor status (10, 13-15, 19-24). The results of some studies are consistent with ours (10, 15, 19, 21, 25) while others are not (13, 14, 22, 24). Prati *et al.* evaluated 200 cases and reported that hormone receptor-positive tumors had a 9.6% incidence of HER2 overexpression, and this rate rose to 31.2% for hormone receptor-negative tumors (10). The evaluation of 134 cases of breast cancer by Okaly *et al.* showed that ER- and PR-negative tumors had 74% and 69% rates of HER2 amplification, respectively (15). Moreover, Toucher *et al.* evaluated HER2 status in 923 patients with breast cancer and found that HER2 overexpression was correlated with negative ER/PR and grade III lesions, and young age (19). In another study on 256 invasive breast cancers, HER2-positive status was significantly associated with ER negativity (21). An association between HER2 overexpression by FISH and ER negativity, PR status, P53 negativity, and high Ki67 labeling index was reported in one study on 100 patients with invasive breast ductal carcinomas (25). Konecny *et al.* in 2003 showed that even when tumors were positive for both hormone receptors (ER, PR) and HER2, the level of ER/PR was lower than those in the tumors that had non-amplified HER2 by FISH (22). In contrast to the previous studies, the study of Shaikh *et al.* in Pakistan on 118 breast cancer patients confirmed the relationship between ER and PR positivity, and HER2 overexpression (24). Moreover, the result of a study by Guo *et al. *showed that 43 out of 139 (30.9%) HER2 borderline cases had positive results using the FISH test and that ER positivity, PR positivity, and tumor grade were three predictive factors that could estimate the probability of positive HER2 results by FISH (14). On the other hand, there is a study on 108 cases of breast cancer that showed no significant association between hormonal receptor status and HER2 status (13).

Like other studies, HER2-positive ILC was very rare in our study. We had 31 ILC patients and only two of them (6.5%) which were of pleomorphic ILC variant showed positive HER2 results by FISH. Our results do agree with others in that most cases of ILC with HER2 overexpression represented the pleomorphic variant (26). Kee *et al. *reported a higher prevalence of HER2-positive classic type ILC (10.8%) compared to the previous ILC case series (1-6%) (27). In the study of Prati *et al.*, only three cases of 31 (9.7%) ILCs out of 200 breast cancer cases had positive HER2 results by FISH (10). Conversely, HER2-positive classic type ILC as a rare entity was strongly associated with the absence of PR expression in another study (28). 

Several studies have revealed the association between poor grades and FISH positivity (10, 19, 21, 23). Evidence on the low probability of FISH positivity in a low-grade tumor is strong enough to convince some researchers that HER2 assessment may be considered unnecessary in a subgroup of low-grade tumors (10, 19, 29). In a study on 177 cases of well-differentiated breast cancers that were HER2-borderline on IHC, the rate of HER2 amplification by FISH was 1.7% (3/177) and all three HER2-positive tumors had low levels of amplification (30). The prevalence of HER2 positivity among patients with well-differentiated tumors was reported from 0% in some studies (21, 24) to less than 5% in other studies (10, 31), and less than 10% in Taucher’s study (19). In the latter, the likelihood of HER2 positivity was 6.1% in hormone-receptor-positive patients with tumor grades I and II (19). Similarly in our study, 13.9% (46 out of 330) of patients with low-grade tumors (I & II) had HER2 amplification by FISH but no statistically significant association was found between the grade and HER2 amplification. However, in our study, the tumor grade had not been mentioned on core biopsy samples in many patients, and the pathologists were not able to determine it on many surgical lumpectomy specimens because of complete or near-complete response to neoadjuvant chemotherapy. The missing information about the grade of tumors in this subgroup of patients might have altered our results.

Our study had some advantages. First, our sample size was large enough to find an association, in contrast with many of previous studies. Since there is a high concordance (96%) between FISH and CISH in the determination of HER2 status in breast cancer reported (32), the results of this retrospective study with two methods are acceptable. The second advantage was that all FISH and CISH tests were done by a dedicated referral laboratory in our country. This study had also an important limitation due to missing data about the grade, and LVI, especially in patients who had undergone neoadjuvant chemotherapy which may alter the results.

## Conclusion

In conclusion, in HER2 borderline breast cancer, the rate of HER2 positivity may show a significant correlation with the tumor size and PR negativity and may show a reverse association with the presence of histologic ILC. In order to find an applicable model or algorithm to predict the FISH/CISH results in HER2 borderline breast cancer in practical pathology, further multi center studies are recommended. Considering the high cost of FISH and CISH, the results of such studies especially in low-income countries such as Iran could prevent treatment delays in patients with breast cancer.

## Conflict of Interest

The authors declared no conflict of interest.
